# Rosacea prevalence, severity, and phenotype by race, ethnicity, and gender: A cross-sectional study of a diverse patient cohort within a primary care population

**DOI:** 10.1016/j.jdin.2025.11.016

**Published:** 2025-11-27

**Authors:** Alexander R. Gomez-Lara, Adam Zakaria, Maria Elena Sanchez-Anguiano, Erin H. Amerson

**Affiliations:** a Kaiser Permanente Bernard J. Tyson School of Medicine, Pasadena, California; b Department of Dermatology, University of California, San Francisco, California; c University of California, Davis School of Medicine, Sacramento, California; d Department of Dermatology, Zuckerberg San Francisco General Hospital, San Francisco, California

**Keywords:** rosacea, severity, skin of color

*To the Editor:* Rosacea presents with several distinct clinical phenotypes, as described in the updated 2019 Global ROSacea Consensus Panel criteria.^1^ Rosacea prevalence and rosacea phenotype prevalence have primarily been studied among White European populations and vary by region and diagnostic method.^2^ A systematic review and meta-analysis estimated subtype prevalence among rosacea patients as 72% to 80% erythematotelangiectatic and 18% to 28% papulopustular, with higher prevalence among women compared to men.^2^ Given limited data describing rosacea prevalence, severity, and phenotypes among US racial/ethnic minorities, we analyzed these characteristics among a cohort of patients within a large, racially/ethnically diverse primary care population.

In this institutional review board-approved cross-sectional study (#20-33109), we reviewed charts of adult patients within the San Francisco Health Network (an affiliated network of primary care clinics) assigned an International Classification of Diseases-10 code for rosacea by primary care or a consulting dermatologist from August, 2019 to April, 2024 (Table I). We included patients with sufficient documentation to determine rosacea phenotype and treatment type offered at first visit (topical vs oral therapy, used to approximate severity) and stratified by self-reported racial/ethnicity (Table II).^1^ Data were collected in RedCap (version 14.5.44), and chi-squared and logistic regression were performed using STATA (version 18.0).

**Table I:** Characteristics of SFHN rosacea patients by race and ethnicity (*N* = 1180)

	Race/ethnicity							*P* values
	Total	Asian	Black or African American	Hispanic	Multiracial/other∗	White	Skin of color†	
Total SFHN population with and without rosacea‡	109,866 (100)	23,015 (20.9)	16,686 (15.2)	38,761 (35.3)	10,404 (9.5)	21,000 (19.1)	88,866 (80.9)	
No. diagnosed with rosacea (%)	1180 (1.2)	192 (0.8)	19 (0.1)	517 (1.3)	95 (0.9)	357 (1.7)	823 (0.9)§	<.01
No. diagnosed by primary care physician (%)‖	672 (57)	118 (62)	10 (53)	337 (65)	56 (59)	151 (42)	521 (63)§	<.01
Age at diagnosis in years, mean (SE)	51.0 (0.4)	54.9 (0.9)	50.8 (3.2)	48.9 (0.6)	45.2 (1.3)	53.4 (0.69)	50.0 (0.49)§	<.01
Sex, no. (%)¶								
Female	730 (61.9)	147 (76.6)	14 (73.7)	342 (66.2)	56 (60.0)	171 (47.9)	559 (67.9)§	<.01
Gender identity, no. (%)‖								
Female	627 (53.1)	129 (67.2)	14 (73.7)	297 (57.5)	41 (43.2)	146 (40.9)	481 (58.4)§	<.01
Male	362 (30.7)	40 (20.8)	4 (21.1)	140 (27.1)	28 (29.5)	150 (42.0)	212 (25.8)§	
Chose not to answer	180 (15.3)	22 (11.5)	1 (5.3)	76 (14.7)	25 (26.3)	56 (15.7)	124 (15.1)§	
Non-Binary	11 (0.9)	1 (0.5)	0 (0)	4 (0.8)	1 (1.0)	5 (1.4)	6 (0.7)§	

*SFHN*, San Francisco Health Network.

∗ Other includes patients that chose American Indian/Alaska Native, Native Hawaiian or Other Pacific Islander, Declined to Answer, Other, Unknown, or None of the Above.

† Skin of color includes Asian, Black or African American, Multi-racial/other, and Hispanic.

‡ Population data from in-network referring clinics from August, 2019 to April, 2024.

§ *P* < .01.

‖ All patients were members of the SFHN primary care population. Some were referred to dermatology at San Francisco General Hospital, the referral center for specialty care within the SFHN, for diagnosis and/or management of rosacea. Provider specialty (primary care vs dermatology) was identified from the International Classification of Diseases-10-associated encounter.

¶ Of patients diagnosed with rosacea.

**Table II:** Adjusted prevalence of morphological features and severity of rosacea by race and ethnicity (*N* = 1180)

	No. (%)	Race/ethnicity							*P* values
		Total	Asian	Black or African American	Hispanic	Multiracial/other∗	White	Skin of color†	
Diagnostic features	Phymatous changes	72 (6.1)	5 (2.6)	2 (10.5)	40 (7.7)	7 (7.2)	18 (5.0)	54 (6.5)	.33
	Persistent erythema	929 (78.3)	158 (82.3)	13 (68.4)	409 (78.5)	73 (75.3)	276 (77.1)	653 (78.8)	.52
	Ocular rosacea	67 (5.6)	18 (9.4)	0 (0)	20 (3.8)	1 (1.0)	28 (7.8)	39 (4.7)‡	.03
Major features	Flushing and/or transient erythema	71 (6.0)	15 (7.8)	0 (0)	37 (7.1)	7 (7.2)	12 (3.4)	59 (7.1)‡	.01
	Papules and pustules	736 (62.0)	104 (54.2)	14 (73.7)	366 (70.3)	55 (56.7)	197 (55.0)	539 (65.0)§	<.01
	Telangiectasias	428 (36.1)	60 (31.3)	1 (5.3)	159 (30.5)	32 (33.0)	176 (49.2)	252 (30.4)§	<.01
Minor features	Burning or stinging sensation of the skin	43 (3.6)	4 (2.1)	0 (0)	30 (5.8)	3 (3.1)	6 (1.7)	37 (4.5)‡	.02
	Dry sensation of the skin	68 (5.7)	22 (11.5)	2 (10.5)	33 (6.3)	5 (5.2)	6 (1.7)	62 (7.5)§	<.01
	Edema	17 (1.4)	0 (0)	0 (0)	13 (2.5)	0 (0)	4 (1.1)	13 (1.6)	.55
Therapy	Topical	817 (68.8)	137 (71.4)	8 (42.1)	331 (63.5)	68 (70.1)	273 (76.3)	544 (65.6)§	<.01
	Oral	370 (31.2)	55 (28.6)	11 (57.9)	190 (36.5)	29 (29.9)	85 (23.7)	285 (34.4)§	

Values were adjusted for age and sex using a logistic regression.

∗ Other includes patients that chose American Indian/Alaska Native, Native Hawaiian or Other Pacific Islander, Declined to Answer, Other, Unknown, or None of the Above.

† Skin of color includes Asian, Black or African American, Multi-racial/other, and Hispanic.

‡ *P* < .05.

§ *P* < .01.

Of 109,866 primary care patients, 1829 (1.7%) had at least 1 International Classification of Diseases-10 code for rosacea; of these, 1180 (65%) met inclusion criteria (61.9% female, mean age 51.0; 30.0% White, 44.0% Hispanic, 2.0% Black, 16.0% Asian, 8.0% Multiracial/other). Estimated prevalence among White patients was greater than skin of color patients (1.7% vs 0.9%, *P* < .01). Six hundred seventy-2 (56.9%) patients were diagnosed by primary care, while 454 (38.6%) were diagnosed by a dermatologist. In aggregate, persistent erythema (78.4%) and papules and pustules (61.8%) were most represented among the cohort. Oral therapy use was higher among skin of color patients compared to White patients (34.4% vs 23.8%, *P* < .01). Additionally, patients with skin of color had significantly higher prevalence of flushing and/or transient erythema (7.1% vs 3.4%, *P* < .01), papules and pustules (64.8% vs 54.9%, *P* < .01), burning or stinging sensation of the skin (4.5% vs 1.7%, *P* < .05), and dry sensation of the skin (7.5% vs 1.7%, *P* < .01) compared to White patients. After controlling for age, gender, and type of diagnosing provider, Black or African American (odds ratio 5.31, 95% CI [2.04, 13.8], *P* < .01) and Hispanic patients (odds ratio 2.04, 95% CI [1.48, 2.81], *P* < .01) had higher oral therapy use compared to White patients.

We found lower overall rosacea prevalence but a higher proportion of oral therapy use among patients with skin of color. While the decision to use topical versus oral therapy may not always correlate with disease severity, oral treatment is recommended for moderate to severe disease^3^ and may be a reasonable approximation. However, precise characterization of disease severity (eg, Rosacea Area and Severity Index^4^ or other severity criteria) could not be reliably estimated from available chart data. Other limitations include single geographic population, high rate of nondermatologist diagnosis, availability of retrospective chart data, and self-reported race/ethnicity, which may lead to misclassification of skin of color. Future research should prospectively explore the unique manifestations of rosacea among people with skin of color to improve diagnosis and treatment strategies.

## Conflicts of interest

None disclosed.
